# Lifestyle Course as an Investment in Perceived Improved Health among Newly Arrived Women from Countries outside Europe

**DOI:** 10.3390/ijerph111010622

**Published:** 2014-10-15

**Authors:** Solvig Ekblad, Ulla-Britt Persson-Valenzuela

**Affiliations:** 1 Cultural Medicine Unit, Department of Learning, Information, Management and Ethics, Karolinska Institutet, SE-171 77 Stockholm, Sweden; 2 Swedish Course for Adult Immigrants. Södertälje Municipality, SE-151 89 Södertälje, Sweden; E-Mail: ulla-britt.persson-valenzuela@sodertalje.se

**Keywords:** lifestyle course, migration, Sweden, Arabic-speaking, empowerment, health literacy, Somali-speaking, women

## Abstract

Family reunification was the most common reason (34%) for resettlement in Sweden in 2013. About one-fifth of the population is foreign-born. This study used mixed methods to evaluate a culturally tailored clinical health-promotion intervention. The intervention was conducted by licensed clinicians and a local coordinator. Sessions were five-weeks long, two hours a week. The quantitative data cover results from 54 participants, mainly Arabic and Somali-speaking, who participated in 10 groups. The participants’ perceived health improved significantly over the three measures. They also shared that their health significantly improved according to moderate effect size. The qualitative data, analyzed using revised content analysis, reflected one general theme: “the intervention is an investment in perceived improved health”, and four categories: “perceived increased health literacy”, “strength, empowerment and security”, “finding a new lifestyle”, and “the key to entry into Swedish society is language”. An intervention focusing on the prevention of ill-health, on health as a human right, and on empowerment, and aimed at female newcomers, has practical implications.

## 1. Introduction

In recent years, human migration has changed, with the process of globalization, in number and nature. Migration is influenced by various factors that include the seeking of asylum and personal safety, the quest for economic improvement, and family reunification [1]. In recent decades, Sweden, which previously had a fairly homogenous population, has become much more culturally diverse [2]. In 2013, according to statistics from the Swedish Migration Board [3], total immigration for the purpose of family reunification was 40,026 (34%), making it the most common reason for immigration among the total of 116,587 new immigrants with permission to stay. This paper focuses on that specific group of migrants. One-fifth of the Swedish population (9,644,864) are foreign-born. Due to the situations in Arabic and Somali-speaking countries, the most common languages of the recent arrivals are Arabic and Somali.

The Swedish Public Health Report (2009) shows that the prevalence of physical and mental ill-health among immigrants is double those of the Swedish-born population [4]. In light of this, there has been a call to integrate migration-related health issues with the health-equity framework [5]. In most countries and settings, women have poorer health than do men, but they outlive men [6]. Further, Wamala and her colleagues tested the theory of intersectionality, including place of birth with gender [7]. The results were not in line with the social-gradient theory: women of foreign background with high income levels were the worst-off category in terms of self-rated health [7]. A register study shows that the relative risk of hospitalization due to depressive disorder following unemployment is highest among immigrant women in Sweden [8]. Still, there is a lack of coordinated policy approaches to address the health implications of modern migration [9]. As our societies are characterized by diversity, more research is required, with a view to empowering migrants to integrate with society, obtain better care and learn to correctly use the healthcare system [10].

According to a theoretical approach by Silove [11], events that elicit stress after arrival in the reception country, that is, “postmigration stress”, have a negative impact on health-related quality of life among newcomers [12]. Presentations of tailored, evidence-based knowledge, an innovative model of culturally tailored health promotion groups for refugees were given in the U.S. [13] and among refugees in Swedish contexts [14,15], with promising results. However, there is a knowledge gap as to how such a lifestyle course addressing recently arrived immigrant women as relatives might promote self-care and proper use of healthcare system. This article focuses on the benefits of such an intervention for newly arrived women from countries outside Europe—that is, how they perceived their health before and after participating in the intervention. The results will be of significance not only for the individual and his or her relatives but also for healthcare and society as a whole.

The research questions were: How do participants assess their health prior to, at the end of, and six months after the end of the lifestyle course? How do the participants view their participation in the course?

This study is part of a larger study designed to assess self-perceived health and well-being among newly-arrived women who immigrated as relatives, before and after they participated in the intervention [16].

## 2. Methods

### 2.1. Description of the Intervention

In this article we use a definition of health similar to that used in earlier publications, which perceive health as “a dynamic, active process of maintaining a personal and social state of balance and well-being” [13,17].Participants are the key agents in promoting their own healing.

This is a participatory approach—that is, the participants are invited to engage in a dialogue with the multidisciplinary team and ask questions about health, lifestyle and prevention. Furthermore, this approach generates a group dynamic that results in peer recommendations, which may be more effective than individual dialogue.

The participants were seated in a classroom setting. Arabic and Somali-speaking women were the most common participants. Each intervention was dedicated to a single language group. The course lasted for five weeks, with a two-hour meeting on the same day and time each week. The last session was extended to 2.5 h to allow for a diploma ceremony for participants who attended at least four of the five sessions. Regarding the curriculum’s pedagogical technique, the multidisciplinary team was sensitive to the participants’ educational level. Each lesson was developed and designed to be clear and simple: the two hours were divided into one for health information, followed by a short break for some free refreshments, and one for dialogue and feedback about the relevance of the information presented. Generally, participants were given a hand-out containing PowerPoint presentations including Swedish-language illustrations, which provided opportunities to learn practical health-related Swedish vocabulary. During the course, the ability to laugh and a sense of humour were an advantage, according to clinical experience [15].

The approach focused on nutrition and public health, physical activity, the female body, contraception and health, stress and coping. The curriculum of the intervention, based on Silove’s theoretical approach, started with focus groups and interviews, by students supervised by the first author, with key local people in order to receive relevant needs content in every session for each language group [18,19,20]. The first session focused on the participants’ personal potential, personalized empowerment, prevention of illness and promotion of health in the shorter and longer term. At every session, the clinical staff in charge described their responsibilities and the educational background they had in Swedish primary care, both in outpatient and inpatient settings. In Table 1, each session is described in more detail.

**Table 1:** The intervention presentation: each session with its objectives, instructional foci, and person in charge of the intervention.

Session 1	Title: Introduction to the Intervention
Content	The first session will establish the atmosphere for the course and will cover an introduction to the course to promote self-care and use of healthcare, and ends with a short relaxation exercise
	In session 1, the local coordinator will Introduce the course and policy Find out (1) what participants know so that the course can build upon this, a dialogue and construct learning, and (2) begin to foster a collaborative, trusting atmosphere within the group and between participants and local coordinator so that open communication will take place throughout the intervention
Intended learner objectives	Participants will: Become acquainted with the theory and philosophy of the course Become acquainted with group members Raise awareness of migration stress and coping
Instructional foci	Lecture and small group discussion, refreshments, relaxation
Person in charge	Local coordinator who is a social worker at the office of Swedish for immigrants (woman)
**Session 2**	**Title: The Health Care Guide (*Vårdguiden*)**
Content	The second session will introduce a nutritional guide with examples of Western and traditional culture material, access to and the organization of the Swedish healthcare system and pharmacies, public health disease prevention and dental care
Intended learner objectives	Participants will: Become acquainted with the nurse profession in primary care Become acquainted with Swedish health care and prevention of common public health diseases Raise awareness about self-care and the “plate model” (*tallriksmodellen*)
Instructional Foci	First hour lecture with PowerPoint presentation Group discussion (e.g., demonstration of sugar in Coca-Cola, sugar reduction for weight loss and sodium reduction for blood pressure control)
Person in charge	Registered nurse in primary care (woman)
**Session 3**	**Title: The Significance of Physical Activity**
Content	The third session will introduce the significance of physical activity for health and includes what happens in the body during physical activity, anatomy (stand up, sit, and lie down), movement, how to lift properly, static/dynamic movement
Intended learner objectives	Participants will Become acquainted with the physiotherapist profession in primary care Become acquainted with the significance to move and rest
Instructional foci	First hour: lecture with PowerPoint presentation Group discussion and exercise (vacuum with weight transfers, lift heavier objects, body movements); ends with reflection
Person in charge	Registered physiotherapist in primary care (woman)
**Session 4**	**Title: Women’s Health and Contraception**
Content	The fourth session will introduce women’s health, gynecological smear tests, mammography, the Swedish abortion law, contraception and pregnancy, menopause, and domestic violence
Intended learner objectives	Participants will Become acquainted with the midwife profession in primary care Become acquainted with women’s health and related issues
Instructional foci	First hour: lecture usually orally presentation Group discussion about own experience of pregnancy and other needs
Person in charge	Registered midwife in primary care (woman)
**Session 5**	**Title: Psychosocial Health**
Content	The fifth session will introduce migration stress related illness, sense of coherence, empowerment, coping, self-care and equity in health
Intended learner objectives	Participants will Become acquainted with social worker as profession in primary care Raise awareness about the concepts of health, stress, sense of coherence, equity in health, and self-care
Instructional foci	First hour: lecture with PowerPoint presentation Group discussion about postmigration stress and coping
Person in charge	Social worker in primary care (woman)

### 2.2. Setting

Our clinical experience is that newcomers may lack a sense of “belonging” with respect to Swedish authorities (e.g., healthcare, social welfare) and may therefore avoid such authorities. To avoid stigma and social isolation, the sessions and follow-ups were held in a neutral place, that is, at the site of the courses in Swedish for adult immigrants in Södertälje. Södertälje is 30 kilometers south of Stockholm. At the end of 2013, Södertälje had a population of 91,072 originating from 40 countries and speaking 80 languages, and about 35 per cent of the population were foreign-born (but held permanent permission to reside in Sweden and came to Sweden for various reasons, or due to their being born in Sweden and both of their parents being foreign-born [21,22].

### 2.3. Invited Participants

Eligibility criteria included being a female new arrival who immigrated to Sweden as a relative within five years after receiving permission to stay in Sweden. According to the EU definition, “new arrival” means having had permission to stay in Sweden for the past five years at least [23]. The local coordinator recruited participants from a convenient cohort of mainly Arabic and Somali-speaking newcomers living in Södertälje. As there was no official introductory immigrant-reception activity for this target group, an official data list on this target group was lacking.

The participants received no monetary compensation for their participation. 

### 2.4. Study Design

This was a prospective study: mixed methods for data collection were used to obtain a better understanding of this research area by combining quantitative and qualitative data [24]. A process evaluator was present in the room to observe and make note of the dynamics of the group during each session and all questions raised. This data will be published elsewhere. The second author and a professional interpreter—who for practical reasons was not always same person—were present at each session. Participation in the survey was voluntary. The participants agreed to participate but were free at any time to terminate their participation in the intervention without prejudice. The second author was in charge of collecting the data on the three occasions, with the help of a professional interpreter. Data consisted of a few questions, which were given before, directly after and at a six-month follow-up. In each group’s first session, the second author went through all the survey questions, giving the participants an opportunity to ask questions about the questionnaire and get answers directly from the second author. Answering the questions took about 10 min.

In the last follow-up we chose to include semi-structured interviews with open-ended questions due to our view, which was based on our experience, that the participants would feel more comfortable responding on the basis of their own narratives. This gave us an opportunity to investigate issues from different perspectives and a foundation from which to confirm the results, which may increase the validity of data [25]. The most recent six-month follow-up took up to one hour.

The project, which started in 28 August 2012 and ended on 30 June 2014, included 277 participants, from Södertälje (145) and Angered (132), which is on the west coast of Sweden close to Gothenburg. In this article we have chosen to report the results of the follow-up survey for the effective participants in Södertälje who attended all three data collections (*n* = 54). The data on 10 groups with five weekly sessions (*n* = about 10 participants/group) were collected from 28 August 2012 to 26 November 2013, as four more groups in Spring 2014 could not be followed up after six months, as the project finished on 30 June 2014. Those who did not respond to the first, second or third follow-up notices are referred to as drop-outs even though they completed the intervention. Other results are reported elsewhere.

### 2.5. Quantitative Data

In order to evaluate the intervention from the participant’s point of view, a user-friendly approach was implemented with each participant during the first and last sessions of the intervention and by meeting each of the 10 groups six months after the intervention. Each participant was given an identity number, participants chose the code themselves and used the same code for all measures (e.g., # or Δ). For practical and language-related reasons we did not use an instrument but included a few questions from Sweden’s national public health survey, “Health on Equal Terms” which also foreign-born had answered before [4]. The validation of these questions primarily involves construct validity, the question's metric capacity to differentiate, previous use of the question and the inherent dropout ratio associated with the question. We asked the following three questions using a 5 Likert Scale: 1. How would you describe your own health? (1 = poor, 5 = excellent); Would you say you have strength and energy? (1 = never, 5 = always) and 3. How much have aches and pains influenced your daily life during the last week? (1 = very much, 5 = not at all). Additionally, we asked about how many hours they slept in general and how often they had nightmares. We also asked about four confidence questions [13]: 1. How confident are you that your health can improve? 2. How confident are you in understanding the causes of your illnesses? 3. How confident are you that you can explain your health problems to your doctor? and 4. How confident are you that your doctor can understand you? The possible answers in those four questions are (1) Not confident; (2) A little confident; (3) Somewhat confident; and (4) Extremely confident.

### 2.6. Qualitative Data at the Six-Month Follow-up

The five main open narrative questions were: (1) If a female relative or friend came to Sweden to live here, what content would be needed for her in a lifestyle course (intervention)? (2) How did you feel before the lifestyle course? (3) How do you feel today? (4) How has the lifestyle course influenced you? and (5) What more do you need to feel integrated in the Swedish society?

### 2.7. Translation/Back Translation

The information, quantitative and qualitative questions were translated into Arabic and Somali by a translator and back-translated orally [26] by an authorized interpreter from Språkservice, with which all public authorities (e.g., Karolinska Institutet) have a contract. These versions were compared for consensus and minor changes of no significance were made.

### 2.8. Data Analysis

Quantitative data acquired from the three measurements were analyzed by producing descriptive statistics using SPSS 22.0 (IPM Corp., New York, NY, USA) for Windows. This analysis included the computing of mean values and standard deviations of all Likert items included. We used Spearman analyses to identify the strengths of associations between ranked (non-parametric) questions. A *t*-test was performed to determine whether the participants showed changes in their perceived health directly after and after six-month follow-up. Effect sizes (Cohen’s *d*) were calculated in order to verify improvement. An effect size of 0.2 is assumed to be small, 0.5 to be average and values over 0.80 to be large. Only *p*-values under 0.05 are reported in the results. Socio-demographic characteristics and lifestyle questions were described by means, standard deviations and frequencies.

An inductive approach was used for the qualitative data, as is advisable when previous knowledge is lacking, which was the case in this study [27]. The data were analyzed using a revised version of a method of qualitative content analysis [28]. Only manifest content was analyzed, as the study relied on an interpreter for translation of communication and analyzing the latent content would therefore be questionable [29]. The answers to the questions and the narratives from the group interview at the six-month follow-up were summarized in notes taken by the second author and independently scored by each of the two authors, who then discussed the coded material and arrived at content categories and themes. Unidentified citations will illustrate the quantitative data.

### 2.9. Ethical Considerations

The study was approved by the Stockholm Regional Ethical Review Board (No. 2012/1302-31/5). Each questionnaire was marked with a code (a number) and this code was used at the measuring dates instead of the participant’s name and could not be linked to how they responded to various questions. The participants were informed in advance, orally and by information sheet, about the purpose of the study and the follow-up. They gave their written or oral (when illiterate) consent to participate and were free at any time to terminate their participation without prejudice. To reduce the risk of exposing an already vulnerable group, the analysis and the presentation of results were performed on the group level and the quotations used are not identified by language group. For ethical considerations, a recorder was not used.

## 3. Results

### 3.1. Participants and Dropouts

The intervention involved six Arabic-speaking groups, two Somali-speaking groups and two groups that spoke several mixed languages. Each of the 10 groups consisted of 3 to 13 participants (Table 2).

A total of 144 female and 1 male participated in the lifestyle course in Södertälje. Of the total 145 participants, 54 answered questions before and directly after and at the sixth-month follow-up, three measures. One hundred and one of the participants answered questions before and directly after. Their responses will be published elsewhere. Forty participants (28.6%) were dropouts for the second and third measures. There were no significant differences regarding age, education level and work experience between the participants who participated in all three measurements and the rest. The typical reasons for dropping out at the six-month follow-up were competing activities, such as introductory courses for employment at the same time and reduction of the accompanying financial support payments if the person did not attend, relocation to another municipality, pregnancy or simply dropping out.

**Table 2:** Number of participants in each group of the 10 Lifestyle courses in Södertälje and with three measures.

Groups (Month)	Language	Number of Participants	Answered Questions during Three Measures
1 (28 August–25 September 2012)	Arabic	3–6	4
2 (2 October–20 October 2012)	Arabic	6–7	7
3 (6 November–4 December 2012)	Arabic	5–9	8
4 (22 January–19 February 2013)	Somali	4–10	4
5 (26 February–26 March 2013)	Somali	3–9	1
6 (2 April–7 May 2013)	Arabic	6–7	5
7 (14 May–11June 2013)	Arabic	5–9	5
8 (20 August–17 September 2013)	Mixed	5–6	6
9 (24 September–22 October 2013)	Arabic	6–13	9
10 (29 October–26 November 2013)	Mixed	9–13	5
**Total**		**52–89**	**54**

Sociodemographic mean data among the 54 participants are presented in Table 3. A little more than half (28%, or 51.9%) were native speakers of Arabic, followed by native Somali speakers (5%, or 9.3%) and native speakers of 12 other languages (21%, or 38.8%). The average age was 34 (8.155), the min–max 20–53, the average number of years in school 9 (4.993), and the school min–max 0–19. Forty-five women (83.3%) were married and 38 (71.7%) had children. Of them, 74% had at least one child (1–5 children) and their children were between 0–29 years old. Before arrival they had worked an average of 5 years (6.825) but it was common that they had been housewives. The main reasons for their coming to Sweden were family reunification (23%, or 44.2%), followed by external stress (20%, or 38.5%), work opportunities (5%, or 9.6%), and other reasons (4%, or 7.7%).

**Table 3:** Sociodemographic data; mean age, years in school, in work and number of children among female participants in three measures in the Lifestyle course in Södertälje (*n* = 54).

Sociodemographic Data	Mean (SD)
Mean age (SD)	34 (8.155)
Min–max	20–53
Mean years in school in home country	9 (4.993)
Min–Max	0–19
Mean years in work before arrival	5 (6.825)
Min–Max	0–28

Table 4 demonstrates the female participants’ self-reported perception of health before, directly after and following the six-month follow-ups. They thought that their health would be significantly improved, at both the first and the last follow-up (*t* = −3.673, *p* < 0.001; −3.236, *p* < 0.002, respectively) and the effect size was moderate. The participants showed significantly higher perceived health during the last follow-up and the effect size was moderate (*t* = −2.760, *p* < 0.008). According to Table 5, there were significant correlations between the three questions “Would you say that your health is?”, “Would you say that you have strength and energy?” and “How much have aches and pains influenced your daily life during last week?”, and also for each of these three questions during the three measurements, respectively (*at least p<.05*).

It was common that the participants slept about 6–8 hours a night on all three measures, but nearly half (26, or 49.1%) had nightmares before the lifestyle course and about the same (25, or 47.2%) had similar amounts direct after the course and at the six-months follow-up, respectively (25, or 47.2%; 26, or 48.1%).

**Table 4:** Perception of health among female participants in a five weeks’ intervention in Södertälje, before, direct after and at six months follow up (N = 54) the whole group of participants.

Questions	M(SD) before (Range)	M(SD) DirectAfter (Range)	M(SD) afterSix Months (Range)	t(*df*) before-Direct after before-6 Months	*Cohen’s d*
1. Would you like so say that your health is?	3.37 (0.896)	3.83 (0.863)	3.76 (0.910)	−1.965(53) *p* < 0.096	0.41
	(2–5)	(2–5)	(2–5)	−2.760(53) *p* < 0.008	0.43
2. Would you like to say that you have strength and energy?	3.43 (0.838)	3.65 (0.914)	3.46 (0.719)	−2.00(53) *p* < 0.051	0.24
	(2–5)	(2–5)	(2–5)	−0.286(53) *p* < 0.776	0.02
3. How much aches and pain has influenced your daily life during the last week?	3.41 (0.901)	3.52 (0.926)	3.44 (1.022)	−0.799(53) *p* < 0.428	0.08
	(2–5)	(2–5)	(2–5)	0.237(53) *p* < 0.814	0.02
4. I think my health will be improved.	2.90 (0.886)	3.41 (0.790)	3.37 (0.853)	−3.673(49) *p* < 0.001	0.40
	(2–4)	(2–4)	(2–4)	−3.236(49) *p* < 0.002	0.36
5. I think I understand why I perceive illness.	3.10 (0.944)	3.38 (0.837)	3.28 (1.045)	−1.613(50) *p* < 0.113	0.21
	(2–4)	(2–4)	(2–4)	−0.598(49) *p* < 0.553	0.13
6. I think I can explain to doctor or nurse how I feel.	3.35 (0.913)	3.48 (0.771)	3.44 (0.752)	−0.868(50) *p* < 0.389	0.10
	(2–4)	(2–4)	(2–4)	−0.573(48) *p* < 0.569	0.07
7. I think my doctor can understand me.	3.41 (0.853)	3.41 (0.901)	3.40 (0.840)	−0.136(50) *p* < 0.892	0.01
	(2–4)	(2–4)	(2–4)	0.136(49) *p* < 0.892	0.01

**Table 5:** Correlations between questions asked during three measurements (Spearman) (*n* = 54).

Questions	1	2	3	1	2	3		1	2	3	4
	Before	Before	Before	Direct after	Direct after	Direct after		After 6 months	After 6 months	After 6 months	
1. Would you like to say that your health is?				0.493 **	0.10	−0.016		0.374 **	−0.021	0.013	
2. Would you like to say that you have strength and energy?	0.403 **			0.531 **	0.585 **	0.244		0.263	0.312 *	0.135	
3. How much aches and pain has influenced your daily life during last week?	0.273 *	0.393 **		0.489 **	0.435 **	0.391 **		0.328 *	0.202	0.329 *	

Notes: * *p* < 0.05; ** *p* < 0.001.

### 3.2. Qualitative Findings at 6-Month Follow-up

There was one general theme “The intervention is an investment in perceived improved health” and four categories: “Perceived increased health literacy”; “Strength, empowerment and security”; “Finding new ways of life”; and “The key to entry into Swedish society the language”.

#### 3.2.1. Perceived Increased Health Literacy

The content of the information provided at the last follow-up after six-months by the participants of the intervention was considered relevant and new for the participants. The participants mentioned that it was important for them as newcomers to have this new knowledge and they pass on this information to family members at home and also in their home country.

“*Great information. I feel better now. I have sought help for my back pain problems*”*.*

“*The information that I should check my breasts. It was good that women should examine themselves. Nutrition information also gave me new information*”*.*

“*It was good information, so I think today that I feel good by focusing on sleep and exercise. Today, I know where I can go to get more information about something happening*”*.*

“*I have learnt a lot from the nurse, who I will call in different situations when needed. Have told my relatives and given the phone numbers (e.g., emergency call, frågeguiden)*”.

#### 3.2.2. Strength, Empowerment and Security

The course gave the participants new perspectives which reduced their perceived stress. They had the strength to deal with their challenges in new ways, they became motivated and they felt more self-confident and energetic—that is, they felt better than before the course. They could cope with future work options, studies and family and could better help their families feel physically better.

“*Before the course I was stressed. I was thinking too much. It would be better to have this lifestyle course at the time when you are new (in the first six months) in Sweden*”*.*

“*I feel good now, but I had mental problems before. I have gained an understanding of my disease. Previously, I had no information about stress*”.

“*I felt bad before the course, but the course made me stop and think about what I could do to help myself*”.

“*That I could raise questions about pregnancy, food and weight. Today I think more about health and exercise*”.

“*I think that the course gave me a* “*mental lift*”*. I’ve gotten better and will feel better mentally*”.

“*I feel good, because I think more about my health and myself. (Have four children) I’ve started a course to lose weight*”.

#### 3.2.3. Finding New Ways of Living

The participants related their new lifestyles to health promotion and health literacy, considered themselves to have developed stronger will-power and felt that the relaxation session meant that “the soul could rest”. They were regularly monitoring their diet and had changed their eating habits in terms of both nutrition and eating times, and were also monitoring their contraception measures and coping strategies. The course resulted in new perspectives, which influenced their lifestyle as they felt their health improved. After the intervention some participants had begun training which later led to traineeships and, eventually, permanent employment in healthcare. They expressed a willingness to learn more.

“*I go on walks, which I did not do before—it is a new habit. Also, the children and the family have been told about what I learned. I do not cook the same food all the time and I try not to sit too long. I have become calmer*”.

“*I’ve developed, got new contacts (meeting other participants), I am thinking more about my body and have become calmer; I study and I now plan special time for the children and the family*”*.*

“*I’m pregnant and feel tired and unwell, but I think about what I learned about pregnancy and relaxation. It was good. I feel more confident now, earlier I was worried. I have had inflammation in a toe. I was worried it was a clot. I feel more confident now and don’t worry*”.

“*I get a lot of exercise. There are different kinds of contraception in Sweden, where I now have access to primary care and ways to promote health*”.

“*Before I had no friends here, but now I feel my life has become more normal. I get together with relatives and friends and I am less scared. I want to succeed with my education (needed for a job) and bringing up my children*”.

#### 3.2.4. The Key to Entry into Swedish Society is Language

The participants discussed how knowing Swedish would better equip them to communicate their symptoms and needs when they seek primary care. Otherwise, misunderstandings could easily occur. To be dependent on an interpreter was not always good because understanding is important if they are going to describe how they feel. Newcomers should be encouraged to learn the language to express their opinions in the new country. They searched for way to interact with Swedes. Additionally, the role of women in Sweden and in their home countries was quite different, which increased demands on them and they wanted to know more about human rights issues.

“*Language is the most important thing. The more we learn, the better we feel. We need more contact with Swedes, but where can we find opportunities? We rush from school to practice and then home and to our children who need to be cared for. There is a lot of pressure on us. In our country all women were housewives*”*.*

“*I live with a Swedish man, but I want to know more about Swedish legislation—for example, about social issues and how agencies work*”.

### 3.3. Additional Ideas for Content for Future Lifestyle Courses

The intervention attracted interest in learning more about gynecological health, breast cancer prevention, parenting, prevention of physical punishment, first aid, weight loss, rights regarding access to healthcare for asylum seekers, information about dental care, relationship problems, mental health, nutrition, and Swedish traditions and habits.

“*…… I’m curious about Swedish traditions. I think it would be interesting to discuss more about the culture clashes and child rearing. I have seen how many people in my culture have difficulty setting limits for their children. It doesn’t need to be wrong, but we need to get to know each other and each other’s habits, have* *discussions. So there can be a balance – getting to know each other is important*”.

“*I want to know more about children’s health and how to talk to them, in a good way (not being angry)*”*.*

“*I also have to learn more about Swedish culture, to help me feel good and get a job. It is also important to get out in nature*”*.*

“*Stress reduction in the relationship between a man and a woman*”.

## 4. Discussion

### 4.1. Discussion of the Results

The present study raises a number of significant questions, and provides implications and recommendations for policymakers, clinical work and research. The results give support to a study [30] among newly arrived immigrant Iraqis in Sweden showing that there is low awareness of health and preventive healthcare in this group, the conclusion being that “more education for immigrants is needed about health and about the Swedish healthcare system to increase their understanding of these topics” [30]. One of the questions raised by this study is how the reception environment might improve health literacy and coping strategies for newcomers.

The results have connection with Silove’s five dimensions model [11]. The main reason for coming to Sweden was family reunification (44.2%) but 38.5% came due to external stress in their home country (Arabic-speaking and Somali-speaking countries). The fact that nearly half of the participants had nightmares could be connected with traumatic life events [11]. It is also important to ask newcomers about the reason for their migration and not mainly about the country they were born in [31]. Feeling secure, the first dimension in Silove’s theory, was discovered in the interviews, which explored how a perception perspective might be in contrast with the law regarding the permission to stay in the country. Feeling secure is a pre-condition of health, and this may influence how people cope. Discussing different coping strategies needed for the target group in a safe space and in dialogue with the clinician as well as the other group participants developed the group’s collective insight and may have improved their health literacy for everyday life. When the participants in a culturally tailored health promotion groups [13] acquire more strategies and alternatives, their flexibility in terms of adopting a healthy lifestyle, and their potential, increase. Attachment, the second dimension, is connected with the family situation. The majority of the participants came to Sweden for marriage and they felt that they had high demands on them as parents and that taking care of their children took more time as they did not have help from their relatives compared to their home country they got help from other relatives. They missed their relatives, especially the female relatives (mothers and sisters). The participants mentioned that they needed more knowledge about to care for their child in Sweden, and more knowledge about child diseases, gynecology and other challenges that can arise. Regarding the third dimension, identity and role function, the participants were mainly housewives, but several of them wanted to study and work in healthcare, and some had already got the opportunity. Psychological acculturation refers to the dynamic process that the new female reunification immigrants experience as they adapt to the new reception culture [32]. They also wanted more support in that no competing activities from other authorities should be scheduled at the same time as the intervention. A formal agreement with local authorities regarding future courses would be recommended. Human right, the fourth dimension, came up during the interviews, as participants wanted every newly arrived immigrant woman to receive an intervention for illness prevention, empowerment and health literacy. The last dimension in Silove’s model, the existential dimension, came up mainly in the participants’ reflections on their health as a basic issue for a meaningful life. Life events such as moving from outside Europe to Sweden may influence health and possibilities for coping and are important to consider in connection with resettlement. Empowerment is knowing the Swedish language, having access to healthcare when needed and having the ability to influence one’s own life. Health promotion groups [13,14,15] such as this intervention, that is, lifestyle courses, may disseminate knowledge and ways of gaining control over one’s own life. Paying attention in a group conversation involving the participants’ own knowledge about health is also a kind of “power leveling”. Altering attitudes is challenging, but facts and information may change the individual’s insight, which will then impact the person’s behavior in a way that improves his or her perceived health and health-related beliefs [33].

The participants were not identified patients and the aim of the intervention was not care-giving. However, this study as well as earlier Swedish studies [14,15] demonstrates the significance of health as a human right for a group at risk of illness and marginalization and also for their relatives (e.g., husbands and children) as the participants told in the six months’ follow up that they pass on this health literacy to family members at home and also in their home country. In keeping with the literature, the data indicate that our target group needs tailor-made and culturally sensitive mental health promotion programs in order to respond to their own health needs. This indicates that the identification of pre- and post-migration stress challenges, such as perceived health, reason for migration and earlier traumatic life events, should be part of an early introduction, and not only psychiatric and somatic care when they have become patients at a later stage. This will not only have a humanistic perspective. At the same time, with a relatively small and preventive investment, the intervention (10.5 hours over five weeks) can accomplish quite rapid changes for both the target group—changes that are sustainable over six months—and society. The group became a social support group, finding that an optimal number of encounters during five weeks may also prevent marginalization and isolation.

### 4.2. Discussion of the Methods

The study has strength in that it is prospective, used established questions from the Public Health Report [4] and culturally tailored health promotion groups [13], and is based on both quantitative and qualitative data, that is, it used partly a mixed-method design as qualitative data was only used at the last follow-up after six months. The qualitative data explored the quantitative data and demonstrated that the lifestyle course was relevant and empowered the participants. The narrative data had face validity with the quantitative results.

The questions used had not been tested for reliability and validity for this target group, as that would have required additional time and data. The main methodological limitations of this study are the absence of a control group and the convenience sample, implying selection bias, because we could not control who came as participants. Therefore, we could not conclude that the significant changes in perceived health were a result of the lifestyle course. The target group was relatively small and involved mainly two languages (Arabic and Somali) and they had different educational background. Moreover, the questions may have been misunderstood, and the fact that participants have had more or less experience in answering questionnaires may have influenced bias of data. Their knowledge of metric distance, equivalence—for instance, distance on an ordinal scale—may vary, depending on factors such as their educational level and the experience they may have had with such questions in their culture. This knowledge is needed when planning the design.

The target group is less settled than the general population, which had impacts on the numbers of dropouts, but the number dropouts was in line with other prospective studies [14,15] and there were no significant differences in background factors between the participants and the dropouts.

A limitation of the qualitative data is that we communicated through a professional interpreter and due to that, could not analyze latent data [34]. The researchers concluded that the qualitative data was saturated—that is, no new information came out from further interviews [28]. The quality of qualitative studies is assessed by its trustworthiness, whereby different criteria should be met [28]. Credibility depended on the methods used to answer the research question/s. In our study a mixed methods approach was used and the qualitative interviews explored individuals’ experience and perceptions [35]. As both authors had long experience of data collection and were familiar with the aim of the study and were engaged in collecting and analyzing the data, their pre-understanding may have been barrier to their perception of other perspectives. Dependability in a study is related to whether there is a thoughtful way to manage changes in data and analysis over time [28]. In our study, the collection of data was performed over 1½ years, which reduced the risk of changes in methods and pre-understanding. The transferability of a study is connected with how the data is applied to other groups and can be clarified by quotation [28]. Comparing the results with earlier research [13,14,15] is problematic, as the course is culturally tailored, due to the participants’ diversity and needs. In our study we have provided several quotations, so readers can draw their own conclusions about transferability to other groups.

## 5. Conclusions

A relatively short and culturally tailored intervention (lifestyle course), with clinically interdisciplinary health professionals, a local coordinator, a process evaluator, and a professional interpreter, strengthens the prerequisites for increased health literacy among new-coming female women from countries outside the EU. Women’s health is a human right and healthy new arrivals will be productive members in the society.

This intervention should be included in a reception community-based health promotion and accompanied by regular evaluation and quality improvement. This intervention should also be introduced to other social and economic disadvantaged groups at risk for illness in the general population.

Even though the study has limitations mentioned above, this short intervention for unidentified patients has sufficient evidence to assert that is promising tool worthy of further implementation.
